# PTEN expression as a predictor for the response to trastuzumab-based therapy in Her-2 overexpressing metastatic breast cancer

**DOI:** 10.1371/journal.pone.0172911

**Published:** 2017-03-02

**Authors:** Daphne Gschwantler-Kaulich, Yen Y. Tan, Eva-Maria Fuchs, Gernot Hudelist, Wolfgang J. Köstler, Angelika Reiner, Carmen Leser, Mohamed Salama, Johannes Attems, Christine Deutschmann, Christoph C. Zielinski, Christian F. Singer

**Affiliations:** 1 Department of Obstetrics and Gynecology, Cancer Comprehensive Center, Medical University of Vienna, Vienna, Austria; 2 QIMR Berghofer Medical Research Institute, Herston, Queensland, Australia; 3 Clinical Division of Oncology, Department of Medicine I and Center for Excellence in Clinical and Experimental Oncology, Medical University of Vienna, Vienna, Austria; 4 Department of Obstetrics and Gynaecology, Wilhelminenspital, Vienna, Austria; 5 Department of Pathology, Sozialmedizinisches Zentrum Ost, Vienna, Austria; 6 Department of Thoracic Surgery, Otto Wagner Hospital, Vienna, Austria; 7 Department of Pathology, Otto Wagner Hospital, Vienna, Austria; University of South Alabama Mitchell Cancer Institute, UNITED STATES

## Abstract

**Background:**

Even though trastuzumab is an effective therapy in early stage Her-2+ breast cancer, 40–50% of advanced Her-2+ breast cancer patients develop trastuzumab resistance. A potential resistance mechanism is aberrant downstream signal transmission due to loss of phosphatase and tensin homologue (PTEN). This study investigated the relationship between the expression of PTEN and trastuzumab response in Her-2 overexpressing metastatic breast cancer patients.

**Methods:**

Between 2000 and 2007, 164 patients with Her-2+ metastatic breast cancer received trastuzumab-based therapy in our institution. We analyzed PTEN status by immunohistochemistry of 115 available tumor tissues and analyzed associations with other histopathological parameters, response rate, progression free survival (PFS) and overall survival (OS) with a median follow-up of 60 months.

**Results:**

Eighty patients were PTEN positive (69.6%) and 35 patients PTEN negative (30.4%). We found a significant association of the expression of PTEN and p53 (p = 0.041), while there was no association with grading, hormone receptor status, IGFR or MIB. We found significantly more cases with progressive disease under trastuzumab-based therapy in patients with PTEN positive breast cancers (p = 0.018), while there was no significant correlation with PFS or OS.

**Conclusion:**

In Her-2-positive metastatic breast cancers, PTEN positivity was significantly associated with progressive disease, but not with PFS or OS.

## Introduction

Overexpression of Her-2 is found in approximately 25% of human breast cancers leading to an aggressive phenotype and poor patient survival [1,2,3,4]. Trastuzumab, a humanized monoclonal antibody against the extracellular domain of Her-2, has been shown to be very effective in combination with chemotherapy for the treatment of early stages [5,6] or metastatic breast cancer [7,8] and even as a single-agent for the later group [9] with substantial decrease in breast cancer recurrence and mortality [10,11,12,13]. However, 40–50% have shown resistance to trastuzumab administered as a single agent [9] or in association with taxanes [10,14] within one year. However, the exact mechanism of the development of trastuzumab resistance is not completely clarified yet.

One of the known mechanisms underlying trastuzumab’s antitumor activity is the downregulation of p185^ErbB2^ and the subsequent inhibition of its downstream PI3K-Akt and MAPK signalling pathways. Molecules located in these pathways are thought to be associated with unresponsiveness to trastuzumab. PTEN (phosphatase and tensin homologue) is a dual phosphatase with membrane localization, which antagonizes PI3K function and inhibits Akt activities and tumor growth [15]. Consequently, PTEN loss leads to hyperactivation of the PI3K pathway and drives tumorigenesis. It has been shown that loss of PTEN, which occurs in about 20–40%, is associated with resistance to trastuzumab-based therapy [16,17,18,19].

Two reported mechanisms how PTEN loss promotes trastuzumab resistance are the transformation of Her-2 positive breast cancer into a triple negative subtype through induction of the epithelial-mesenchymal transition (EMT) [20] and the development of autophagy defects [21].

Nevertheless, the results of existing studies looking at the role of PTEN expression in the development of trastuzumab resistance are conflicting [22].

We therefore investigated the role of PTEN as prognostic marker in 115 metastatic Her-2 positive breast cancer patients who underwent trastuzumab-based therapy in the palliative setting, and then examined PTEN status and its association with clinical and histopathological parameters such as hormone receptor status, p53, MIB-1, IGFR, grading and clinical outcome (response rate, progression free survival (PFS), overall survival (OS)).

## Materials and methods

### Study population

Between March 2000 and March 2007, 164 patients with metastatic Her-2 grade 2+ or 3+ overexpressing breast cancer received trastuzumab (Herceptin^®^, Roche Pharmaceuticals, Vienna, Austria) with or without chemotherapy at our institution.

Before initiation of trastuzumab-based treatment, all patients had and were required to have bi-dimensionally measurable disease (with both diameters > 1.0cm and at least one lesion with both diameters > 1.5cm excluding CNS lesions as the only site of measurable disease) with clearly defined margins and radiologically (CT and/or MRI and/or ultrasound) documented tumor progression. In accordance with the Southwest Oncology Group response criteria and endpoint definitions [23], response evaluation was performed by independent review of patients’ records and radiology reports. This study was approved by the ethics committee of the Medical University of Vienna and patients had to sign an informed consent prior to inclusion into the study.

Of 164 patients with Her-2 positive metastatic breast cancer who received trastuzumab, 115 formalin fixed paraffin embedded tumor tissues were available for this study. Tumor grade, age at diagnosis and histopathological parameters such as Her-2 status, estrogen receptor (ER), progesterone receptor (PgR), p53, MIB-1 and insulin like growth factor receptor (IGFR) were extracted from clinical records.

Of these 115 patients, 102 patients (88.7%) were Her-2 grade 3+ positive and 13 patients (11.3%) were Her-2 grade 2+ positive with confirmed Her-2 amplification by fluorescence in situ hybridization (FISH). Her-2 grades 2+ or 3+ overexpression was assessed by immunohistochemistry (IHC) according to our institutional practice (as determined by the Herceptest, DAKO Diagnostics, Austria), with confirmation of Her-2 amplification by FISH for all IHC 2+ cases.

### Immunohistochemical detection of PTEN expression

PTEN IHC analyses for this study were conducted using UltraVision LP Detection System. In brief, 4-μm formalin-fixed paraffin embedded tumoral tissue sections were deparaffinised and rehydrated. For antigen unmasking, sections were pre-treated by digestion with Target Retrieval Solution Citrate pH 6 (DakoCytomation). Endogenous peroxidase activity was blocked by incubation in hydrogen peroxide block. Sections were then blocked with Protein Block (DAKO X0909) and incubated with the primary mouse monoclonal anti-PTEN antibody (Clone 6H2.1, Cascade Biosciences, diluted 1:300) over night at 4°C. After incubation with the secondary anti-mouse IgG antibody (Vector Laboratories cat. # PK6102) for 30 min and washing with PBS buffer, slides were incubated with ABC reagent for 30 min at room temperature. The immunoreaction was visualized by using Liquid DAB-PLUS Substrate Kit (Zymed Laboratories; cat# 00–2020) according to the manufacturer’s protocol. Slides were then counterstained with haematoxylin and cover-slipped.

**Fig 1:**
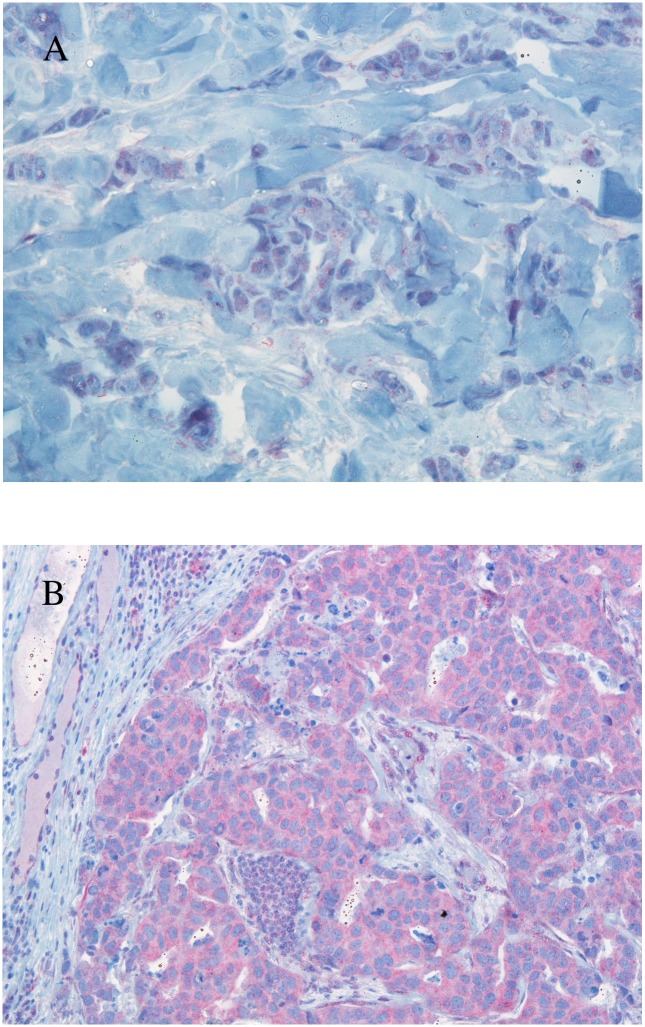
Example of immunohistochemical detection of PTEN: A: Negative PTEN expression (IRS 1); B: Positive PTEN expression (IRS 2).

In all subjects, invasive area, in situ area and surrounding breast tissue were separately evaluated for presence of PTEN antibody. Cells were rated as showing nuclear staining or cytoplasmic staining for PTEN. Due to the variations in the intensity of staining, a scoring system was used [16]. The percentage of cells that were semi-quantitatively positively stained for PTEN and the intensities of staining were multiplied and the result was recorded as immunoreactive score (IRS). Intensities of staining were graded as 0 (negative), 1 (weakly positive), 2 (moderately positive) and 3 (strongly positive). Staining was graded as 0 (<5% cells), 1 (5–25% cells), 2 (26–50% cells), 3 (51–75% cells) and 4 (>75% cells). Based on these results, IRS 0–3 was recorded as 0, IRS 4–6 as 1 positive, IRS 7–9 as 2 positive and IRS 10–12 as 3 positive. PTEN positivity was defined as IRS 2 or 3 and PTEN negativity as IRS 0 or 1. Negative control slides without primary antibody were included for each staining. Normal tonsil epithelium known to express normal PTEN was used as positive control. Expression levels were assessed independently by two experienced pathologists from two different institutions (A.R. and J.A). Photomicrographs of immunohistochemical analysis for PTEN expression are depicted in Fig 1.

### Statistical analysis

Chi Square and Fisher’s Exact test were used to evaluate associations between PTEN expression and clinicopathological parameters. Logistic regression was used to predict if any of the clinicopathological parameters predicts PTEN positivity. Complete, partial remission, stable and progressive disease were defined by World Health Organization (WHO) criteria [24].

Progression free survival was defined as the time from the start of trastuzumab-based treatment to disease progression or death. Overall survival (OS) was defined as the time from initial diagnosis of breast cancer (OS1), from the occurrence of metastatic disease (OS2) and from the start of therapy with trastuzumab (OS3) to death from any cause. Median years for PFS and OS and 95% confidence interval (95%CI) were determined for all patients and were estimated according to the Kaplan-Meier product limit method. Log rank test was used to compare survival distributions of patients with PTEN positivity versus patients with PTEN loss.

For all analyses, a p-value <0.05 was considered statistically significant. SPSS statistical software system (SPSS Inc., Chicago, IL, version 23.0) was used for all calculations.

## Results

### Study population

A total of 115 patients with Her-2/*neu* overexpressing metastatic breast cancer, who received trastuzumab-based treatment at our institution between March 2000 and March 2007 were included in this study. Patients, tumor characteristics and types and lines of trastuzumab-based therapy are shown in Table 1. These patients were followed up for a median observation period of 60 months (range 1 to 168 months), during which 38 (33%) objective responses were observed (including 16 complete responses (13.9%) and 39 (33.9%) patients experienced disease stabilization (≥ 3 months, with stable disease lasting ≥ 6 months in 8 patients). Thirty-four patients (29.6%) experienced progressive disease under trastuzumab-based therapy. Overall, 104 patients (90.4%) had experienced disease progression and 98 (85.2%) deaths had occurred, all of which were attributed to disease progression. Eight patients with complete response experienced disease progression after a median period of time of 10.6 months (range 6 to 27 months) after completion of trastuzumab-based therapy. No patient was lost to follow-up. Median progression-free survival (PFS) (from the beginning of trastuzumab-based therapy until progressive disease) was 6.3 months (95% CI -1.77 to 44.78). Median overall survival from diagnosis of breast cancer (OS1) was 5.0 years (95% CI 1.0 to 18.0), from diagnosis of metastatic disease (OS2) 3.0 years (95% CI 0–14) and from the start of trastuzumab-based therapy (OS3) 2.0 years (95%CI 0–10), respectively (Table 2).

**Table 1:** Patients and tumour characteristics, including type and line of trastuzumab-based therapy for these patients in the metastatic setting.

Characteristics	*N (%) Total = 115*
Mean age at diagnosis (range)	51.1 (25–82)
Menopausal status	
Premenopausal	50 (43.5)
Postmenopausal	65 (65.5)
Grading	
G1	4 (3.5)
G2	21 (18.3)
G3	87 (75.7)
Unknown	3 (2.6)
Hormone receptor status	
ER+/PgR+	15 (13.0)
ER+/PgR-	24 (20.9)
ER-/PgR+	3 (2.6)
ER-/PgR-	65 (56.5)
Unknown	8 (7.0)
Dominant site of metastatic disease	
Bone	58 (51.8)
Visceral	77 (66.4)
CNS	7 (6.1)
Lymph nodes	64 (55.2)
Trastuzumab therapy	
Alone	13 (11.3)
With hormonal therapy	7 (6.1)
With chemotherapy	89 (77.4)
Taxane	19 (16.5)
Vinorelbine	62 (53.9)
Xeloda	5 (4.3)
Taxane+Anthracycline	3 (2.6)
Unknown	6 (5.2)
Lines of trastuzumab therapy	
First line	80 (69.6)
Second line	24 (20.9)
Third line	7 (6.1)
Unknown	4 (3.5)

Abbreviations: ER = estrogen receptor, PgR = progesterone receptor, CNS = central nervous system

**Table 2:** Clinical outcomes of 115 metastatic breast cancer patients.

Clinical outcomes	N (%)
Assessable patients for response	111 (96.5)
Objective response	
CR	16 (13.9)
PR	22 (19.1)
SD	39 (33.9)
PD	34 (29.6)
Unknown/missing	4 (3.5)
Median TTP, months (range)	6.3 (-1.77–44.78)
Median overall survival, months (range)	
From diagnosis of breast cancer (OS1)	5.0 (1–18)
From diagnosis of metastatic disease (OS2)	3.0 (0–14)
From the start of trastuzumab (OS3)	2.0 (0–10)

**Abbreviations:** CR = complete response; PR = partial response, SD = stable disease, PD = progressive disease; TTP = time to progression from the beginning of trastuzumab-based therapy; OS = overall survival: OS1 = from diagnosis of breast cancer, OS2 = from diagnosis of metastatic disease, OS3 = from the start of trastuzumab.

### PTEN expression and its association with other tumor characteristics and clinical outcomes

Distribution of histopathological characteristics by immunohistochemistry of 115 breast cancer specimens is shown in Table 3. PTEN expression was positive in 80 (69.6%) and negative in 35 (30.4%) cases. Looking at the PTEN expression status and possible associations with other immunohistochemical tumor characteristics, we found a statistically significant association between PTEN positivity and p53 (p = 0.041, Chi-square test). There was no significant association between PTEN expression and hormone receptor status, MIB-1 or IGFR (Table 4).

**Table 3:** Tumor characteristics by immunohistochemistry of 115 breast cancers.

Immunohistochemical characteristics	*n (%)*
PTEN status	
Neg	35 (30.4)
Pos	80 (69.6)
ER status	
Neg	68 (59.1)
Pos	39 (33.9)
Unknown	8 (7.0)
PR status	
Neg	89 (77.4)
Pos	18 (15.7)
Unknown	8 (7.0)
P53 status	
Neg	42 (36.5)
Pos	56 (48.7)
Unknown	17 (14.8)
MIB-1 status	
Neg	27 (23.5)
Pos	51 (44.3)
Unknown	37 (32.2)
IGFR	
Neg	21 (18.3)
Pos	19 (16.5)
Unknown	75 (65.2)

Abbreviations: PTEN = phosphatase and tensin homologue; ER = estrogen receptor; PR = progesterone receptor; MIB-1 = Molecular Immunology Borstel; IGFR = insulin like growth factor receptor

**Table 4:** PTEN expression and its association with histopathological parameters and clinical outcome.

	PTEN		Chi-square	Fisher´s	Logistic regression, univariate		Multivariate regression	
	Neg (total = 35)	Pos (total = 80)	p-value		OR (95%CI)	p-value	OR (95%CI);	p-value
	N(%)	N(%)						
ER status								
neg	17 (54.8)	51 (67.1)	0.23		1.0			
pos	14 (45.2)	25 (32.9)			0.60(0.25–0.60)	0.23		
PR status								
neg	24 (77.4)	65 (85.5)	0.31		1.0			
pos	7 (22.6)	11 (14.5)			0.58 (0.20–1.67)	0.31		
P53								
neg	17 (58.6)	25 (36.2)	**0.04**		1.0			
pos	12 (41.4)	44 (63.8)			2.49 (1.03–6.05)	**0.04**	2.62(1.04–6.59);	**0.041**
MIB-1								
neg	9 (36.0)	18 (34.0)	0.860		1.0			
pos	16 (64.0)	35 (66.0)			1.09 (0.40–2.96)	0.86		
IGFR								
neg	4 (57.1)	17 (51.5)	0.79		1.0			
pos	3 (42.9)	16 (48.5)			1.26 (0.24–6.50)	0.79		
Response								
Complete	4 (12.1)	12 (15.4)		0.12	1.0			
Partial	11 (33.3)	11 (14.1)			0.33(0.08–1.36)	0.13		
Stable	8 (24.2)	31 (39.7)			1.29 (0.33–5.10)	0.72		
Progress	10 (30.3)	24 (30.8)			0.80 (0.21–3.09)	0.75		
Progressive disease								
No	8 (23.5)	6 (7.6)	**0.02**		1.0			
Yes	26 (76.5)	73 (92.4)			3.74 (1.19–11.81)	**0.02**	4.40 (1.21–15.99);	**0.02**
Median OS, years (range)			Log-rank					
			p-value					
OS1	5.0 (3.3–6.7)	5.0 (3.5–6.5)	0.99					
OS2	2.0 (1.1–2.69)	3.0 (2.3–3.7)	0.90					
OS3	2.0 (1.0–3.0)	2.0 (1.1–2.8)	0.94					

Abbreviations: PTEN = phospatase and tensin homologue; ER = estrogen receptor; PR = progesterone receptor; IGFR 1 = insulin like gowth factor 1; OS = overall survival: OS1 = from the diagnosis of breast cancer, OS2 = from the diagnosis of metastatic disease, OS3 = from the start of trastuzumab-based therapy.

While we did not observe any statistically significant difference regarding objective response rates to trastuzumab based therapy (ie. complete response (CR) and partial response (PR)) and rates of clinical benefit (CR, PR and stable disease (SD)) between patients with tumors expressing PTEN as compared to those with PTEN loss, we found a significant association between PTEN positivity and progressive disease (p = 0.018). Log rank test did not show any significant difference in PFS or OS between patients with tumors expressing PTEN and those with PTEN loss (Table 4)

## Discussion

Although trastuzumab is the mainstay of therapy in Her-2 overexpressing breast cancer, some patients do not respond. While there are many possible mechanisms leading to trastuzumab resistance, the most important one is activation of the PI3K/Akt signal pathway. The key tumor suppressor gene that limits the activation of this pathway is PTEN. Nagata et al first showed that PTEN loss, which has been reported in 35–86% of Her-2-positive breast cancer, causes unresponsiveness to trastuzumab [16]. Nevertheless, studies have reported conflicting results regarding prediction of trastuzumab response by PTEN and other elements of the PI3K/Akt pathway: PTEN expression and status of PI3K mutation are predictive of trastuzumab response only if evaluated together leading to activation of PI3K/Akt signalling and disease progression under trastuzumab based therapy [25–27]. In contrast, other studies have reported no association of PTEN, Akt and PI3K with response to trastuzumab-based therapy, neither alone nor when evaluated combined as co-expression [22,28,29]. Two studies showed a correlation of PTEN positivity with more advanced clinical stage of breast cancer without predictive value for the outcome of trastuzumab-based treatment in the neoadjuvant [30], adjuvant and metastatic setting [31].

Furthermore, there are also controversial results regarding the prognostic value of PTEN and other molecules of the PI3K-Akt pathway in breast cancer in the current literature: On one hand, there are studies that found no association between PTEN and PI3K and survival [28, 32–34]. On the other hand, other study groups have reported that loss of PTEN and PI3K mutation are poor prognostic factors in breast cancer and associated with shorter PFS and OS [26,35–39]. Okutur et al reported shorter OS in patients with PTEN-/p27- and PTEN-/Akt- breast cancers while PTEN loss alone was not predictive of shorter survival [40].

Because of these heterogenous results we conducted the present study and looked at the predictive and prognostic value of PTEN in Her-2 positive metastatic breast cancer patients treated with trastuzumab- based therapy with a long term follow up of median 60 months.

In our series of 115 metastatic breast cancer patients we have shown a similar distribution to previous studies with PTEN positivity in 69.6% of cases and loss of PTEN expression in 30.4% of cases [18,22,28,32,35,41].

Regarding the predictive and prognostic value of PTEN expression, we have found a statistically significant association of PTEN positivity with progressive disease under trastuzumab- based therapy, but not with PFS or OS.

When we analyzed associations of PTEN with other histopathological parameters, we found a significant positive association of PTEN positivity with p53. In contrast to our results, other studies have reported that high expression of p53 is associated with PTEN loss in basal-like tumors [41,42]. There are no studies showing an association of p53 and PTEN expression in Her-2 positive disease. When we looked at other immunohistochemical parameters like hormone receptor status, MIB or IGFR, we did not find a significant association with the expression of PTEN, which is in line with previous reports [31,32].

Although our study is limited by the heterogeneity of therapeutic approaches in this relatively small sample size and by the fact that we concentrated on PTEN expression without additional information about the PI3K mutation status, we have shown a predictive value of PTEN positivity for the response to trastuzumab- based therapy in metastatic breast cancer patients with a long term follow up of five years. Our results encourage further investigation to define the role of PTEN in the pathogenesis of breast cancer and its role in trastuzumab resistance in larger trials.

In conclusion, we observed more cases with disease progression under trastuzumab-based therapy in patients whose breast cancers expressed PTEN. This underlines the predictive value of PTEN expression for the response under trastuzumab-based therapy. Further studies are warranted to validate these results to be able to further individualize Her-2-targeted therapy and its combinations with PI3K inhibitors and divide this group of patients with aggressive disease as early as possible in responders and non responders.
